# Association between Body Composition and Dysphagia in Patients with Amyotrophic Lateral Sclerosis

**DOI:** 10.3390/neurolint13030032

**Published:** 2021-07-19

**Authors:** Cristina Salvioni, Adriana Leico Oda, Marco Orsini, Michele Pauli, Luciana M. Frabasile, Percilia C. L. Alves, Rosana M. Borges, Helena N. M. Sierra, Gabriela Menegatti, Márcio Ottoboni Pinho, Acary Souza Bulle Oliveira

**Affiliations:** 1 Department of Clinical Neurology, Federal University of Sao Paulo, 04023-900 Sao Paulo, Brazil; adrileico.oda@uol.com.br (A.L.O.); michelepaulli@yahoo.com.br (M.P.); lucianamf@me.com (L.M.F.); perci.cla@gmail.com (P.C.L.A.); roborges8@uol.com.br (R.M.B.); helenanoemi@yahoo.com.br (H.N.M.S.); gabimenegatti@gmail.com (G.M.); marcio@ottoboni.com.br (M.O.P.); acary.bulle@unifesp.br (A.S.B.O.); 2 Department of Medicine, Iguacu University—UNIG and Master Program in Neurology—Vassouras University—USS, 28300-000 Rio de Janeiro, Brazil; orsinimarco@hotmail.com

**Keywords:** amyotrophic lateral sclerosis, nutrition, body composition, deglutition disorders

## Abstract

Background: The influence of changes in body composition on swallowing in patients with Amyotrophic Lateral Sclerosis (ALS) is unknown. Understanding the interrelation between body compartments and dysphagia may establish specific treatments related to both nutritional aspects as to myofunctional ones designed to delay swallowing loss. Aim: The aim of the study was to evaluate the relationship between body composition and dysphagia during the course of the disease. Methods: The protocol of this study included assessments carried out quarterly for one year and included: analysis of body composition by multi-frequency segmental bioimpedance, nutritional diagnosis, maximum strength test of the tongue and lips, analysis of swallowing using the Functional Oral Intake Score (FOIS) and Swallowing Rating Scale of the American Speech-Language-Hearing Association (ASHA), speech intelligibility and analysis of disease severity. To measure the degree of relationship between quantitative variables, Spearman’s correlation was used. Results: Thirty-four patients were evaluated, 28 Spinal Group and 6 Bulbar Group. The results did not show any significant differences in the analysis of body composition between the groups. Positive associations were found between body compartments and swallowing analysis. The phase angle showed a strong correlation between the FOIS scales (r = 0.74, *p* < 0.01), ASHA (r = 0.77, *p* < 0.01) and tongue (r = 0.66, *p* < 0.01). Conclusions: Changes in body compartments were related to swallowing functionality and speech intelligibility in ALS patients, emphasizing the importance of analyzing body compartments for decision making by the interdisciplinary team. Although these preliminary data were collected in a small sample size, they serve to motivate future studies in this area.

## 1. Introduction

Amyotrophic Lateral Sclerosis (ALS) is a progressive degenerative disorder of the upper and lower motor neurons, causing weakness and wasting of muscles controlling limb movement, speech, swallowing and breathing. Eventual respiratory failure and malnutrition with dehydration are the primary cause of death [1]. Worldwide incidence ranges from 1.5 to 2.7:100,000 people/year [2].

ALS is presented in two main different forms: bulbar progressive paresis, (bulbar onset, 25–35% of patients) or spinal motor neuron injury (limb onset or peripheral onset) [3,4,5]. Almost 80% of ALS patients with bulbar onset will develop dysarthria and dysphagia. In a spinal or peripheral onset of the disease muscle weakness is the main symptom [1]. Mean survival of ALS is 3–5 years, with 5–10% living longer than 10 years [3].

During the course of the disease, a progressive decline in muscle function depends on both motor neuron degeneration and nutritional status. Malnutrition is an independent prognostic factor for survival [1,6,7,8,9]. Changes in the body compartments of these patients, regardless of weight reduction, with loss of fat-free mass were also associated with shorter survival [10], just as the increase in body fat has been identified as a protective factor in the evolution of the disease [7,11]. Desport et al. (2003) [12], validated the use of Bioelectrical Impedance (BIA) to assess body composition in ALS patients. Until today, the analysis of body composition has not been used frequently in clinical practice to monitor the progression of the disease and guide the conduct of the interdisciplinary team.

Nutritional aspects and swallowing are interrelated. Dysphagia, caused by weakness resulting from the flaccidity or spasticity of the muscles of the oropharyngeal region, is one of the most serious and debilitating symptoms of the disease [13] and it is the major cause of changes in the nutritional status of patients with ALS [14]. Understanding the interrelation between body compartments and dysphagia may establish specific treatments related to both nutritional and myofunctional aspects designed to delay swallowing loss. In the present study, therefore, we aim to assess the relationship between body composition and dysphagia during the course of the disease.

## 2. Methods

### 2.1. Study Participants

This is a longitudinal and observational study, with a convenience sample recruited from the Neuromuscular Diseases Research Sector at the Federal University of São Paulo. Patients diagnosed with ALS were included, following the criteria defined by El Escorial Criteria from the World Federation of Neurology [15], in regular follow-up with a team of nutritionists and speech therapists, therefore following the same medication protocol proposed by the institution’s medical team, without distinction between groups. Furthermore, patients who had an alternative feeding route at the time of the first evaluation, tracheostomy and who had a history of other neurological impairments were excluded. The study was approved by the institution’s Research Ethics Committee, under number 0288/2017.

Participants were divided into two groups, according to the form of the disease manifestation: Spinal Group (SG), with onset of the disease by upper or lower limbs, and Bulbar Group (BG), beginning with speech, swallowing or atrophy and tongue fasciculation and facial weakness.

The protocol of this study included nutritional assessment and aspects of swallowing performed quarterly for a period of one year, so that the follow-up could take place in four assessments carried out between the times (T1, T2, T3 and T4).

### 2.2. Nutritional Assessment

The patients were weighed on a platform scale (Toledo do Brasil) with a capacity of 500 kg. The height was estimated through the leg length, according to the equations proposed by CHUMLEA et al., 1985 [16] for patients who did not walk or evaluated with the individual standing with the aid of a fixed digital stadiometer HM-210D, with a 1 mm register (InBody Ottoboni^®^, HM-210D, InBody Co., Ltd., Seoul, Korea). The Body Mass Index (BMI) was calculated according to the formula BMI = weight/height^2^ and classified according to age [17,18]. The body composition assessment was performed with the patient in the supine position on a stretcher with a non-conductive surface using the InBody S10 multi-frequency segmental bioimpedance device (InBody Co., Ltd., Seoul, Korea), Ottoboni^®^. The measures evaluated by the BIA were: Body Fat Mass (BFM), Percent Body Fat (% BF), Visceral Fat Area (VFA), Fat Free Mass (FFM), Skeletal Muscle Mass (SMM), FFM of Trunk (t FFM) and Phase Angle (PA).

### 2.3. Swallowing Aspects

To obtain pressure and resistance measurements of the phono-articulatory structures, the Iowa Oral Performance Instrument (IOPI) was used [19]. The device consists of an air bulb connected to a pressure transducer and the values are displayed on the screen, measured in kPa. With the patient seated, instructions were given to measure the maximum pressure of the structures-lips and tongue. The bulb was placed in three positions: tongue on the anterior part against the anterior portion of the hard palate, and on the right and left labial commissures with the instruction to press as hard as possible. Each movement was performed three times with time intervals of 30 s and considered the largest measure. To assess the change in the individual’s functional swallowing and/or the ability to swallow over time, the Functional Oral Intake Score (FOIS) [20] and Swallowing Rating Scale of the American Speech-Language-Hearing Association (ASHA) [21] were used. Speech intelligibility was classified, according to the Yorkston proposal [22].

### 2.4. Disease Progression

The assessment of disease progression was performed using Amyotrophic Lateral Sclerosis Functional Rating (ALSFRS-R). The scale was analyzed by its total score (ALSFRS-R Total) and its subscale scores: bulbar subscale score, fine motor subscale score, gross motor subscale score and respiratory subscale score. ALSFRS-R total scores range from 0 to 48, with 48 indicating no impairment and 0 being most impaired; ALSFRS-R subscales scores range from 0 to 12 with 12 indicating no impairment and 0 being most impaired [23].

### 2.5. Statistical Analysis

The Shapiro–Wilk normality test was applied to continuous variables. As for longitudinal analyzes, that is, for comparison between follow-up times, Friedman’s nonparametric tests (when comparing all times) and Wilcoxon (when comparing only 2 times-T1 and T4)) were used. To measure the degree of relationship between quantitative variables, the Spearman’s correlation. The value of statistical significance adopted was *p* < 0.05 (bicaudal). Correction of significance was performed using the False Discovery Rate method (FDR). FDR is a method of conceptualizing the type I error rate in null hypothesis tests when conducting multiple comparisons. The analysis was performed with the aid of the statistical program Statistical Package for Social Science, version 26.0.

## 3. Results

Thirty-four patients diagnosed with ALS were included in the study, the categorical clinical variables are shown in Table 1.

**Figure 1:**
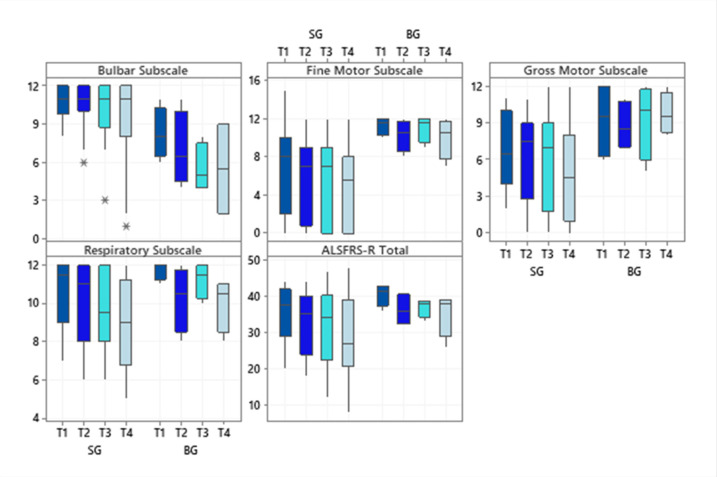
Boxplots of the subscale scores and the total score of the Amyotrophic Lateral Sclerosis Functional Rating Scale-Revised at each time evaluated, according to the two diagnostic groups. Abbreviations: ALSFRS-R (Amyotrophic Lateral Sclerosis Functional Rating Scale–Revised); SG (Spinal Group); BG (Bulbar Group). Significance determined using the Mann–Whitney test; * *p* < 0.05.

Regarding the investigated subscale scores of the ALSFRS-R scale, SG patients had higher scores for the subscale bulbar (*p* = 0.02 at T1 and *p* = 0.04 at T4) while BG patients showed significantly higher scores on the fine motor subscale (*p* = 0.03 in T1 and T4). Even though the BG showed higher scores for the gross motor subscale at all times evaluated, only a statistically significant difference was observed in the final evaluation (*p* = 0.01). The respiratory subscale and the total score did not differ significantly between the diagnostic groups (Figure 1).

**Figure 2:**
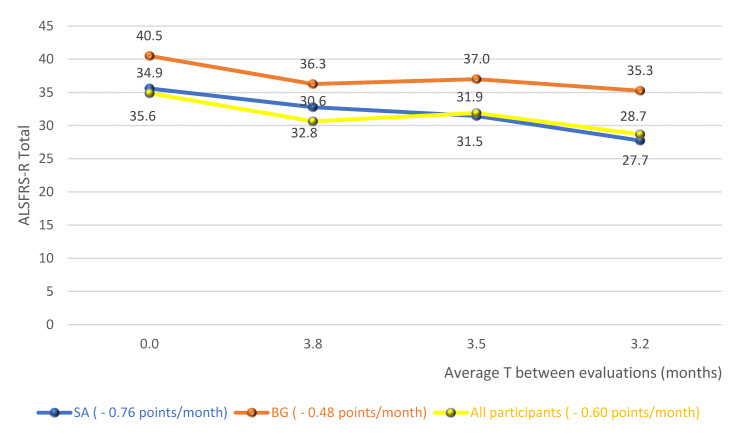
Averages of the total score of the functionality scale for the diagnostic groups and the whole cohort studied. Abbreviations: ALSFRS-R (Amyotrophic Lateral Sclerosis Functional Rating Scale–Revised); SG (Spinal group); BG (Bulbar group).

Figure 2 shows the averages of the total score of the functionality scale for the diagnostic groups and the whole cohort studied, without distinction of the diagnosis, over the follow-up period. In this analysis, there is, therefore, an average fall of 0.76 points/month for SG, 0.48 points/month for BG and 0.60 points/month for the whole cohort.

The classification of the nutritional status, according to the BMI, of patients at the time of assessment (T1) and at the end of the study (T4), is shown in Table 2. At the beginning of the research, there is a higher frequency of malnutrition among patients in BG compared to SG. On the other hand, at the end of the study, malnutrition remained constant in BG and there was an increase in SG.

**Figure 3:**
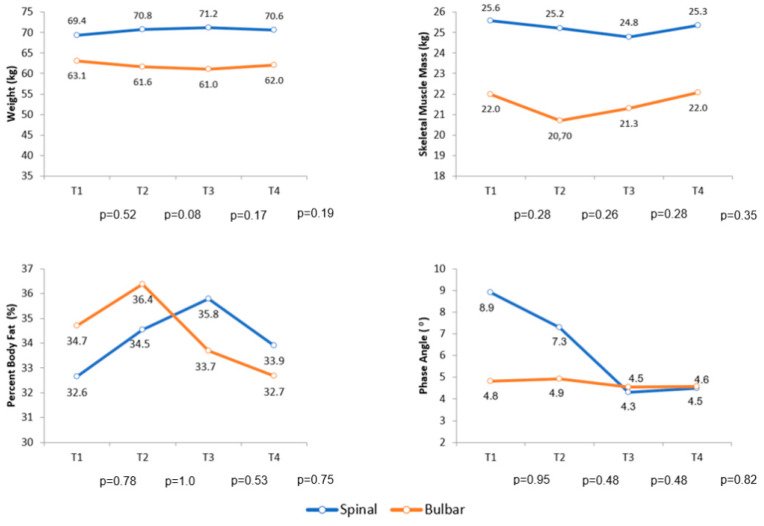
Average variation in body weight, skeletal muscle mass, percent body fat and phase angle during follow-up. Abbreviations: T (Follow-up time).

Over the follow-up period, the comparison of body composition by BIA of the diagnostic groups did not show a statistically significant difference for any variable analyzed in this research. Figure 3 shows the variation in body weight, skeletal muscle mass, percent body fat and phase angle during follow-up.

**Figure 4:**
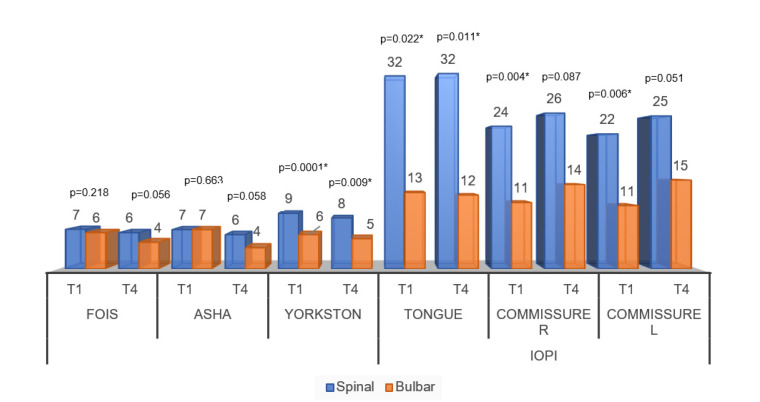
Averages of the comparisons of speech-language assessment measures between the diagnostic groups. Abbreviations: FOIS (Functional Oral Intake Scale); ASHA (Swallowing Rating Scale of the American Speech-Language-Hearing Association); Speech intelligibility by Yorkston); Commissure R (Commissure right); Commissure L (Commissure left); IOPI (Iowa Oral Performance Instrument). Significance determined using the Mann–Whitney test; * *p* < 0.05.

Figure 4 shows the comparisons of speech-language assessment measures between groups. For the temporal analysis of the behavior of the FOIS and ASHA scale, there were no significant differences between the moments of the initial and final evaluations. Thus, there was no difference between the scales that sought to assess the functionality of swallowing for the groups. In the same graph, significant differences can be noticed for all variables of pressure and speech intelligibility scale.

The following analyzes sought to assess the association between body composition and clinical speech therapy assessment. For the SG, the FOIS and ASHA scales showed a significant positive association for BMI and body weight, while the Yorkston scale only for weight. Significant correlations were observed, directly proportional, between tongue pressure and right and left labial commissure and also weight, FFM, SMM and t FFM. In the BG, the FOIS, ASHA scales and tongue pressure showed significant correlations with the BP. For the SG, without the FDR, we obtained 16 rejected null hypotheses and with the FDR, 13 hypotheses. In BG, without FDR, 3 null hypotheses rejected and with FDR, 2 hypotheses. Thus, as there were 54 analyses, the proportion of SG decreased from 29.6% to 24.1% whereas BG decreased from 5.6% to 3.7% (Table 3).

For the SG, the clinical speech therapy findings were positively correlated with all subscales and for the total health of the ALSFRS-R. The correlation in the BG showed a direct association between the FOIS and ASHA scales and the bulbar and respiratory subscale scores. Regarding the pressure of phono-articulatory organs, only the right commissure showed a direct correlation with the total score; (r = 0.54) (*p* < 0.05). For the SG, without the FDR, we obtained 29 rejected null hypotheses and with the FDR, there were no changes in the analyses. For BG, without FDR, 5 null hypotheses rejected and with FDR 2 hypotheses. Thus, as there were 30 analyses, the proportion in SG remained at 96.7% and in BG, it decreased from 16.7% to 6.7% (Table 4).

## 4. Discussion

The results did not show significant differences in the analysis of body composition between the groups. Positive associations were found between body compartments and swallowing analysis. The demographic characterization of the sample agrees with the worldwide distribution reported in epidemiological studies [5,24].

The ALSFRS-R was evaluated not only by its total score, but also by its subscale scores. In the scales where there is a sum of points, it was observed that the total score does not reflect the particularity of each subscale, which becomes very relevant, depending on the analysis that is intended to be conducted [25,26]. When observing the differences between the groups evaluated with the subscale scores of the ALSFRS-R, as evidenced by the characteristic itself of the disease, bulbar change was more frequent and more severe in the BG, while changes in motor function initially compromised the SG.

In the analysis of the variation in the total score of the scale, over the follow-up period, it was possible to observe a fall of 0.76 points/month for the SG, 0.48 points/month for the BG and 0.60 points/month for the whole cohort (Figure 2). A study that provided a detailed description of the clinical trial database at ALS (PRO-ACT), in order to answer important questions about the natural history of the disease, observed a functional decline rate of 1.02 points/month of ALSFRS-R [27]. A recent study, controlled placebo and double blind, with lipid supplementation, found a reduction of 0.69 points/month in the supplemented group [28]. Given these results, the big question was: what would have caused the reduction in the scale found in this research to have been similar or less than that of controlled clinical trials? It is possible that the regular and systematic treatment carried out by an interdisciplinary team with expertise in the treatment of ALS has contributed to the achievement of such results [29,30]. However, a possible sample selection bias, with patients presenting the slowest evolution of the disease, could also have contributed to the achievement of these results.

Although the disease with bulbar onset can be considered a greater risk of malnutrition, in this study this was not proven. If inadequate caloric intake and weight loss are correlated with the severity of dysphagia [31,32], it is possible that the increased energy expenditure due to spasticity and fasciculations be a determining factor for nutritional risk also to occur with patients with onset appendicular [6]. Still, it is possible that the nutritional status of those studied was influenced by gastrostomy, more present in BG [33].

The findings of the swallowing functionality evaluated by the FOIS scale, are in line with the results found by ALVES et al. (2018) [34], in which the index obtained was lower for patients with bulbar onset of the disease. Like the FOIS scale, the functional measures of swallowing obtained by the ASHA scale also declined insignificantly over time. Because it is an evolutionary disease, in which the signs and symptoms appear progressively, patients develop strategies that help them maintain functionality, regardless of their muscular condition. Such strategies may involve swallowing maneuvers, the use of facilitative measures, the choice of certain utensils that favor the grasping and accommodation of food and also management of the environment, such as the elimination of distracting factors. Thus, it is observed that the functionality of swallowing and the adaptation of food consistency for meals remain stable in the analysis of the scales adopted, since the compensation used by patients is not considered in their analysis.

Speech intelligibility differentiated the groups from each other. The process of denervation of the bulbar musculature results in deterioration and eventual loss of the ability to swallow and speak. Therefore, dysarthria and dysphagia are common symptoms in patients with ALS [35], and they can coexist above all in patients with involvement of the bulbar musculature. At some point, communication is affected during the course of the disease [36], which would explain at the end of the study, the changes of speech having also marked the SA. Thereafter, it was possible to observe that, although the neuroanatomy functional basis is the same, the clinical evolution of dysarthria does not present the same linearity.

As for the pressure measurements of the evaluated structures, as in this study, other studies have also shown differences in tongue pressure, differentiating the evaluations [37,38]. In a recent study, ninety percent of patients with dysphagia had an average tongue pressure of less than 34.2 kPa [38]. The results obtained in the T4 of this research, showed impairment in the prominent swallowing for the BG. The decrease in tongue strength at the time of diagnosis has already been identified as an independent prognostic factor for the survival time in patients with ALS [37]. The increase of 1 kPa in the tongue pressure presented by the patients led to a 6% reduction in the probability of indicating PEG [39] elements that would justify the more striking presence of the alternative feeding route in the BG. As well as tongue pressure measurements, lip pressure measurements also showed a significant reduction with the worsening of ALS [39].

Positive associations were observed between the parameters analyzed in the speech therapy assessment and body composition. The justification of the relationship between the findings is based on the alignment of body segments and their biomechanics, whereas changes in body compartments, especially measures related to muscle mass, could compromise such structures. Axial muscle weakness can lead to postural changes in the trunk, considered to be the region of all static compensations, for having connected to it, the upper and lower limbs and the cervical [40]. In addition, the limb weakness itself could misalign this central axis, leading to two clinical conditions: changes in the cervical axis, which can directly interfere with the functioning of the structures connected to it, and the reduction of the base support, making it difficult for the muscles that have the action of pressure valves, as is the case of the lips and the soft palate [41,42]. Such placements would help justify the positive associations found between the measures of the evaluation of swallowing to skeletal muscle mass, free mass and fat and, mainly, fat free mass of the trunk, for SG. Still, the neck muscles contribute to the stabilization and protection of the cervical spine and, therefore, any impairment of these muscles could also cause problems in the swallowing process [43]. In ALS, a prospective and multicenter cohort study demonstrated that weakness of the neck flexors is a potent factor for predicting survival and for worsening activities of daily living, such as speech, swallowing, upper limb function, turning on bed and even walking [44]. Weakness in the neck as an initial symptom was also associated with a shorter survival time for these patients [45].

The loss of muscle mass associated with dysphagia has been established in other studies [46,47,48,49,50]. The decrease in skeletal muscle mass has been identified as a potential risk factor for dysphagia in sarcopenic patients [48]. Lip strength and tongue strength are also associated with sarcopenic dysphagia and are useful indexes for rehabilitation [51]. Wakabayashi et al. (2017) [50], assessed the prevalence of skeletal muscle mass loss and its association with swallowing function in patients with dysphagia after cardiovascular surgery. In the results, it was possible to observe that the prevalence of loss of skeletal muscle mass in these patients was very high and independently associated with the function of swallowing, after adjustment for tracheostomy tubes.

The analysis of the PA brought interesting results. The PA evaluated by the total body analysis was positively associated with the FOIS, ASHA scales and with the tongue pressure, but only for the BG. It must be determined, under the analysis of new studies, whether the PA will also be effective in verifying changes in swallowing for patients who start the disease by limbs. A study that evaluated the multi-dimensional electrical impedance myography of the tongue as a potential biomarker for ALS, observed that the PA, specifically of this organ, had lower values in the patients evaluated when compared to healthy adults [51].

The clinical measures chosen for the speech-language assessment of this study, despite the limited correlations found between the ALSFRS-R and the BG, proved to be relevant for monitoring the progression of the disease. The limited number of patients in the BG may have contributed to these results. As well as the results presented here, other studies have also found correlations between the same speech therapy measures used here with the ALSFRS-R [52,53].

Changes in body compartments were related to swallowing functionality and speech intelligibility in ALS patients. Although the results presented improve the understanding of the contribution that body composition could have in the loss of swallowing and dysphagia, being able to cause nutritional decline, the study is limited by its small sample size. However, the interrelation between nutritional aspects and swallowing deserves more attention in order to motivate further prospective studies in this area. Still, analyzing nutritional decline through the systematic assessment of the body composition of patients with ALS could assist in the clinical management of dysphagia, helping the decision making of the interdisciplinary team.

This study was approved by the relevant research ethics committee and informed consent was obtained from the research subjects.

## Figures and Tables

**Table 1:** Categorical clinical variables of subjects.

Demographic Data		N	(%)		
Diagnosis	■ Spinal onset	28	82.4		
	■ Bulbar onset	6	17.6		
Gender	Men	19	55.9		
	Woman	15	44.1		
Age (Years)	<60	15	44.1		
	≥60	19	55.9		
	**n (T1)**	**n (T1)**	**(%)**	**n (T4)**	**(%)**
NIV	Yes	1	2.9	11	32.3
PEG	Yes	0	0	6	17.6

Abbreviations: Y (Years); NIV (Non-Invasive Ventilation); PEG (Percutaneous Endoscopic Gastrostomy); T1 (Baseline); T4; (Final evaluation); N (Number of patients).

**Table 2:** Classification of the nutritional status, according to the BMI, according to diagnostic groups.

	Spinal Group				Bulbar Group			
BMI	N (T1)	% (T1)	N (T4)	% (T4)	N (T1)	% (T1)	N (T4)	% (T4)
Malnutrition	1	3.6	4	14.3	1	16.7	1	16.7
Normal	18	64.3	15	53.6	4	66.6	3	50.0
Overweight	4	14.3	3	10.7	1	16.7	2	33.3
Obesity	5	17.8	6	21.4	0	0	0	0

Abbreviations: BMI (Body Mass Index); T1 (Baseline); T4; (Final evaluation); N (Number of patients).

**Table 3:** Spearman’s correlation coefficients between body composition and clinical speech therapy assessment, according to diagnostic groups.

Spinal Group										
		BW	BMI	BFM	%BF	VFA	FFM	SMM	t FFM	PA
FOIS	Corr (r)	**0.223**	0.175	0.023	0.010	0.116	0.099	0.086	0.017	0.079
	*p*-value	0.030 *	0.256	0.910	0.935	0.473	0.592	0.657	0.920	0.659
ASHA	Corr (r)	0.170	**0.329**	0.033	0.076	0.153	0.168	0.157	0.127	0.076
	*p*-value	0.256	0.008	0.893	0.659	0.300	0.256	0.292	0.411	0.659
Yorkston	Corr (r)	0.034	**0.288**	−0.031	0.094	0.110	0.205	0.197	0.181	0.072
	*p*-value	0.893	0.050	0.896	0.657	0.592	0.253	0.256	0.289	0.732
Tongue	Corr (r)	0.089	**0.420**	**0.242**	−0.058	−0.009	**0.451**	**0.434**	**0.442**	0.136
	*p*-value	0.657	0.001	0.025 *	0.769	0.935	0.001	0.001	0.001	0.411
Commissure R	Corr (r)	0.073	**0.402**	−0.180	−0.019	0.012	**0.364**	**0.336**	**0.315**	0.038
	*p*-value	0.693	0.001	0.256	0.920	0.935	0.004	0.008	0.014	0.893
Commissure L	Corr (r)	0.137	**0.396**	−0.137	0.031	0.059	**0.337**	**0.314**	**0.312**	0.041
	*p*-value	0.411	0.001	0.411	0.893	0.769	0.008	0.014	0.014	0.891
**Bulbar Group**											
		**BW**	**BMI**	**BFM**	**%BF**	**VFA**	**FFM**	**SMM**	**t FFM**	**PA**
FOIS	Corr (r)	0.362	0.309	0.199	0.292	0.306	0.295	0.324	0.402	**0.741**
	*p*-value	0.509	0.509	0.543	0.509	0.509	0.509	0.509	0.509	0.001
ASHA	Corr (r)	0.313	0.253	0.171	0.238	0.260	0.255	0.281	0.335	**0.777**
	*p*-value	0.509	0.509	0.618	0.509	0.509	0.509	0.509	0.509	0.001
Yorkston	Corr (r)	0.147	0.128	0.271	0.243	0.250	−0.120	−0.103	−0.145	−0.230
	*p*-value	0.670	0.678	0.509	0.510	0.509	0.690	0.706	0.670	0.515
Tongue	Corr (r)	0.370	0.229	0.121	0.157	0.210	0.124	0.164	0.336	**0.660**
	*p*-value	0.509	0.550	0.697	0.670	0.595	0.697	0.670	0.509	0.004	
Commissure R	Corr (r)	−0.284	−0.072	−0.378	−0.265	−0.251	0.301	0.242	0.162	0.136
	*p*-value	0.509	0.777	0.509	0.509	0.515	0.509	0.515	0.670	0.678
Commissure L	Corr (r)	−0.346	−0.099	−0.419	−0.295	−0.289	0.323	0.263	0.145	0.075
	*p*-value	0.509	0.723	0.509	0.509	0.509	0.509	0.509	0.678	0.777

Abbreviations: FOIS (Functional Oral Intake Scale); ASHA (Swallowing Rating Scale of the American Speech-Language-Hearing Association); Yorkston (Speech intelligibility by Yorkston); Commissure R (Commissure right); Commissure L (Commissure left); BW (Body Weight); BMI (Body Mass Index); BFM (Body Fat Mass); %BF (Percent Body Fat); VFA (Visceral Fat Area); FFM (Fat Free Mass); MME (Skeletal Muscle Mass); t FFM (Fat Free Mass of Trunk); PA (Phase Angle); Spearman’s correlation coefficients (Corr (r)). Significance as determined by the Spearman correlation method. * Uncorrected *p*-values for FDR method.

**Table 4:** Spearman’s correlation coefficients between speech-language clinical findings and the total score of the ALSFRS-R and its subscale scores for the diagnostic groups.

Spinal Group						
		ALSFRS-R				
		Bulbar	Fine Motor	Gross Motor	Respiratory	Total Score
FOIS	Corr (r)	**0.568**	**0.258**	**0.289**	**0.401**	**0.420**
	*p*-value	0.000	0.013	0.006	0.000	0.000
ASHA	Corr (r)	**0.588**	**0.277**	**0.277**	**0.406**	**0.427**
	*p*-value	0.000	0.009	0.009	0.000	0.000
Yokston	Corr (r)	**0.664**	**0.437**	**0.257**	**0.209**	**0.465**
	*p*-value	0.000	0.000	0.028	0.074	0.000
Tongue	Corr (r)	**0.411**	**0.491**	**0.310**	**0.284**	**0.491**
	*p*-value	0.000	0.000	0.006	0.010	0.000
Commissure R	Corr (r)	**0.412**	**0.439**	**0.399**	**0.284**	**0.502**
	*p*-value	0.000	0.000	0.000	0.009	0.000
Commissure L	Corr (r)	**0.362**	**0.484**	**0.360**	**0.290**	**0.504**
	*p*-value	0.001	0.000	0.001	0.009	0.000
**Bulbar Group**						
		**ALSFRS-R**				
		**Bulbar**	**Fine motor**	**Gross motor**	**Respiratory**	**Total score**
FOIS	Corr (r)	**0.801**	−0.091	−0.397	**0.500**	0.178
	*p*-value	0.000	0.907	0.374	0.021 *	0.801
ASHA	Corr (r)	**0.822**	0.036	−0.308	**0.546**	0.268
	*p*-value	0.000	0.954	0.473	0.013 *	0.540
Yokston	Corr (r)	0.149	−0.325	0.021	0.276	−0.040
	*p*-value	0.842	0.473	0.954	0.540	0.954
Tongue	Corr (r)	0.403	−0.024	−0.359	−0.081	−0.136
	*p*-value	0.453	0.954	0.473	0.954	0.875
Commissure R	Corr (r)	0.016	0.407	0.262	−0.126	0.544 *
	*p*-value	0.954	0.453	0.652	0.875	0.144
Commissure L	Corr (r)	−0.046	0.181	0.183	−0.157	0.379
	*p*-value	0.954	0.837	0.837	0.842	0.453

Abbreviations: ALSFRS-R (Amyotrophic Lateral Sclerosis Functional Rating Scale–Revised); MMSS (Membros Superiores); MMII (Membros Inferiores); FOIS (Functional Oral Intake Scale); ASHA (Swallowing Rating Scale of the American Speech-Language-Hearing Association); Yorkston (Speech intelligibility by Yorkston); Commissure R (Commissure right); Commissure L (Commissure left); Spearman’s correlation coefficients (Corr (r)). Significance as determined by the Spearman correlation method. * Uncorrected *p*-values for FDR method.
